# TADreg: a versatile regression framework for TAD identification, differential analysis and rearranged 3D genome prediction

**DOI:** 10.1186/s12859-022-04614-0

**Published:** 2022-03-02

**Authors:** Raphaël Mourad

**Affiliations:** CNRS, UPS, MCD, Centre de Biologie Intégrative (CBI), University of Toulouse, 31062 Toulouse, France

**Keywords:** Chromatin interaction, Hi-C, ChIP-seq, Insulator binding protein, Generalized linear model

## Abstract

**Background/Aim:**

In higher eukaryotes, the three-dimensional (3D) organization of the genome is intimately related to numerous key biological functions including gene expression, DNA repair and DNA replication regulations. Alteration of 3D organization, in particular topologically associating domains (TADs), is detrimental to the organism and can give rise to a broad range of diseases such as cancers.

**Methods:**

Here, we propose a versatile regression framework which not only identifies TADs in a fast and accurate manner, but also detects differential TAD borders across conditions for which few methods exist, and predicts 3D genome reorganization after chromosomal rearrangement. Moreover, the framework is biologically meaningful, has an intuitive interpretation and is easy to visualize.

**Result and conclusion:**

The novel regression ranks among top TAD callers. Moreover, it identifies new features of the genome we called TAD facilitators, and that are enriched with specific transcription factors. It also unveils the importance of cell-type specific transcription factors in establishing novel TAD borders during neuronal differentiation. Lastly, it compares favorably with the state-of-the-art method for predicting rearranged 3D genome.

**Supplementary Information:**

The online version contains supplementary material available at 10.1186/s12859-022-04614-0.

## Introduction

In higher eukaryotes, chromosomes are packed into three dimensions (3Ds) and form complex structures [1]. Such 3D structure of chromosomes has recently been investigated by chromosome conformation capture combined with high-throughput sequencing technique (Hi-C) at an unprecedented resolution [2–4]. Hi-C experiments revealed multiple levels of genome organization including compartments A/B [5] and topologically associating domains (TADs) [2, 3]. Most notably, TADs are relatively constant between different cell types and are highly conserved across species. Those TADs play central roles in key cell processes such as for the long-range regulation of genes by enhancers [4] or for the replication-timing regulation [6].

Over the past years, tremendous efforts have been made to develop methods for TAD identification from Hi-C data [7]. The methods can be broadly classified into 4 categories: linear score, statistical model, clustering and network features [7]. The first methods split the genome into bins and define a linear score (insulation score) associated to each bin [2, 8–10]. The second methods rely on statistical models of the interaction distributions [11–13]. The third methods cluster regions of the genome [14–16]. The fourth methods consider the Hi-C data as a graph adjacency matrix and TADs as communities to detect [17–19]. However, very few methods were developed to detect differential TADs between experiments [20–22]. Moreover, few methods were also proposed to predict the impact of chromosomal rearrangement in reshaping TADs, and more generally the 3D genome [21, 23–26].

We propose a versatile regression framework that generalizes the insulation score by estimating a relative score and adding a sparsity constrain (“Sparse Insulation Model”, SIM), but also allows differential TAD analysis (“Differential Insulation Model”, DIM) and Hi-C data prediction after chromosomal rearrangement (“Prediction Insulation Model”, PIM). The proposed model provides a rigorous statistical framework for modeling the interaction distribution, where model parameters represent sparse insulation scores that have an intuitive interpretation and are easy to visualize. Our model assumes additivity of insulation parameters as previously proposed by [24, 25, 27, 28]. By adding interaction terms into the model, the regression framework can naturally be used for differential TAD border identification between two different Hi-C experiments. Moreover, the regression can predict Hi-C data in the case of chromosomal rearrangements such as deletion and inversion, thereby allowing to explore the deleterious impact of de novo enhancer-promoter interactions on genetic diseases and cancers.

Using recent high resolution human and mouse Hi-C data, we found that our approach ranked among the top TAD callers, when evaluated using external assessment designed not to favor any tool. Moreover, it identified new features of the genome we called TAD facilitators, which were demonstrated to be biologically relevant. Our approach could also identify numerous novel TAD borders emerging during cortical neuron differentiation. Such borders were depleted in CTCF compared to embryonic stem cells and enriched in a large number of known neuronal transcription factors including NFATC1/3, NEUROD2, HiC1 and Dmbx1. Lastly, our approach outperformed state-of-the-art algorithm PRISMR to predict Hi-C data after chromosomal rearrangement.

## Materials and methods

### Hi-C data

We used publicly available Hi-C data of lymphoblastoid GM12878 and lung IMR90 cells from Gene Expression Omnibus (GEO) accession GSE63525 [9]. We also used publicly available Hi-C data of mouse embryonic stem (ES) and cortical neuron (CN) cells from GEO accession GSE96107 [29]. Hi-C data were binned at 25 and 50 kb resolutions and normalized by matrix balancing [30].

### Capture Hi-C data

We used publicly available capture Hi-C data of wild-type (WT) and mutant distal limb buds of E11.5 mice from Gene Expression Omnibus (GEO) accession GSE92294 [23]. Hi-C data were binned at 10 kb resolution and normalized by matrix balancing [30].

### ChIP-seq data

We used publicly available binding peaks of 73 chromatin proteins (Rad21, CTCF, YY1, ZBTB33, MAZ, JUND, ZNF143, EZH2, ATF2, ATF3, BATF, BCL11A, BCL3, BCLAF1, BHLHE40, BRCA1, CEBPB, CFOS, CHD1, CHD2, CMYC, COREST, E2F4, EBF1, EGR1, ELF1, ELK1, FOXM1, GABP, IKZF1, IRF4, MAX, MEF2C, MTA3, MXI1, NFATC1, NFE2, NFIC, NFKB, NFYA, NFYB, NRF1, NRSF, P300, PAX5, PBX3, PML, POL2, POL3, POU2F2, RFX5, RUNX3, RXRA, SIN3A, SIX5, SMC3, SP1, SPI1, SRF, STAT1, STAT3, STAT5, TBLR1, TBP, TCF12, TCF3, TR4, USF1, USF2, WHIP, ZEB1, ZNF274, ZZZ3) of GM12878 cells from ENCODE [31]. We downloaded peaks that were uniformly processed (Uniform Peaks).

We also used publicly available CTCF ChIP-seq data of mouse embryonic stem (ES) and cortical neuron (CN) cells from GEO accession GSE96107 [29].

### JASPAR motifs

To scan the mouse genome for motif occurrences, we used FIMO with default parameters (meme-suite.org). The motif position weight matrices were downloaded from JASPAR database (http://jaspar.genereg.net/).

### TAD manual annotation

We used manual annotation of GM12878 TADs at 50 kb from Dali and Blanchette [32]. As previously described by Dali and Blanchette, TADs were manually traced on GM12878 Hi-C maps from the full data set at 50 kb resolution for regions 40-45 mb of 10 different, randomly chosen, chromosomes (chr2, chr3, chr4, chr5, chr6, chr7, chr12, chr18, chr20 and chr22). Briefly, interaction maps of the regions of interest were plotted using HiCplotter. In Adobe Illustrator, dotted squares were manually traced around visually identifiable TADs on the interaction map plots. Regions annotated as TADs had the following properties: (i) sharp visual contrast between within and across TAD interaction frequencies, over the entire TAD region; (ii) minimum size of 250 kb. To give all tools an equal chance, Dali and Blanchette created a dense set of TAD annotations that included any identifiable TAD structure. For example, if two potential TADs were overlapping, both were retained, irrespective of whether one had stronger visual support than the other. TAD boundaries were allowed to overlap or be nested, as long as there is a clearly traceable square along the diagonal. Bed files with TAD ranges were manually created and used for tool comparison.

Since $29\%$ of genomic bins could be considered as relevant TAD borders using this annotation, we considered as TAD borders those supported by at least two TADs that were manually identified.

### Insulation score

For a bin $i \in \{1,...,p \}$, the insulation score was defined as [8]:

1 $$\begin{aligned} IS_i = \log _2 \left( \frac{M_i}{\frac{1}{p}\sum _{i=1}^p M_i} \right) , \end{aligned}$$

where $M_i$ was the number of Hi-C counts that occurred across bin *i* (up to some distance) on the same chromosome.

### Sparse insulation model (SIM)

We first removed the distance effect (polymer effect) from the normalized Hi-C counts using a generalized additive model with a negative binomial distribution:

2 $$\begin{aligned} \log \big ( \rm{E} \big [ \mathbf{y } | \mathbf{d } \big ] \big ) = \beta _0 + f(\mathbf{d }) \end{aligned}$$

Variable $\mathbf{y }$ denoted normalized Hi-C count for any pair of bins on the same chromosome. The log-distance variable **d** accounted for the background polymer effect. The local power law decay relation between distance and Hi-C count was modeled by regression spline [33]. We noted that if bias variables such as GC content, mappability and fragment length were added to the model [34], then the model could also handle unnormalized Hi-C data. Regression residuals (noted **z**) were then used as input for a linear model. Using residuals allowed us to then use best subset selection (L0 penalty) for which there is only linear model implementation in R (see as follows).

Then, a linear model called the “sparse insulation model” (SIM) was proposed to estimate the insulating effects of genomic loci on long-range interactions:

3 $$\begin{aligned} \rm{E} \big [ \mathbf{z } | \mathbf{X } \big ] = \beta _0 + \mathbf{X } \varvec{\beta }_X \end{aligned}$$

Variable set $\mathbf{X }=\{\mathbf{x }_1,...,\mathbf{x }_p \}$ represented the *p* insulation variables, one for each bin of the chromosome. For a bin $i \in \{1,...,p \}$, the insulation variable $\mathbf{x }_i$ was set to one when the bin lied in-between the two bins whose interaction counts were measured by Hi-C, and was set to zero otherwise. The corresponding $\beta _{x_i}$ parameter value reflected the effect of the bin *i* on Hi-C counts. A negative beta value ($\beta _{x_i}<0$) revealed an insulation effect on long-range contacts. Conversely, a positive beta value ($\beta _{x_i}>0$) showed a facilitating effect on contacts. A null beta value ($\beta _{x_i}=0$) meant that the bin had no effect on contacts.

Best subset selection was used to select the best insulation variables when estimating the $\varvec{\beta }_X$ parameters by adding an L0 penalty:

4 $$\begin{aligned} \min _{\beta _0,\varvec{\beta }_X} \frac{1}{N} \sum _{j=1}^N l(z_j,\beta _0 + X_j \varvec{\beta }_X) + \uplambda ||\varvec{\beta }_X||_0 \end{aligned}$$

as done using the L0Learn R package (https://cran.r-project.org/web/packages/L0Learn). Parameter $\uplambda$ was obtained by 10 fold cross-validation of the mean square error (L0Learn.cvfit function with default parameters).

Often the number of insulation variables was too big for L0Learn R package (>5000) and we had to prefilter the variables. For this purpose, we used lasso regression (glmnet R package, https://cran.r-project.org/web/packages/glmnet/) and kept variables with $|{\hat{\beta }}_{x_i}|>0.2$. This allowed to reduce the number of variables to few thousands for L0Learn to work, while still keeping most relevant variables. We found that prefiltering yielded betas that were similar to the ones obtained without prefiltering (Additional file 1: Figure S1).

### Differential insulation model (DIM)

The model could be extended to identify differential TAD borders between two different Hi-C experiment matrices (*e.g.* between two conditions). For this purpose, we first ran SIM for each Hi-C experiment matrix independently. Only the union of bins with $|{\hat{\beta }}_{x_i}|>0$ from both SIMs were kept for differential analysis (we noted the new bin set $\mathbf{S }=\{\mathbf{s }_1,...,\mathbf{s }_q \}$). To prevent bin uncertainty between experiments, only one bin was kept among two consecutive bins. Bins from $\mathbf{S }$ were then used to build a novel model for differential analysis called the “differential insulation model” (DIM).

The differential insulation model was written as follows:

5 $$\begin{aligned} \rm{E} \big [ \mathbf{z } | \mathbf{S },\mathbf{e } \big ] = \beta _0 + \mathbf{S } \varvec{\beta }_S + \beta _e\mathbf{e } + \sum _{j=1}^q \beta _{s_{j}e}\mathbf{s }_j \mathbf{e } \end{aligned}$$

Variable $\mathbf{e }$ denoted the experiment from which the Hi-C count is measured. Variable $\mathbf{s }_j \mathbf{e }$ was the interaction term between the insulation variable $\mathbf{s }_j$ and the experiment variable $\mathbf{e }$, computed as the product between both variables. For a bin *j*, a negative beta value ($\beta _{s_{j}e}<0$) revealed higher insulation effect on long-range contacts for the 2nd experiment compared to the 1st experiment, while a positive value ($\beta _{s_{j}e}>0$) meant lower insulation effect. A null value ($\beta _{s_{j}e}=0$) showed no differential effect. Because the model used as input only bins previously identified by the sparse insulation model, there was no need to use any penalty for parameter estimation. Moreover, the absence of a penalty term allowed to estimate differential effects without bias.

### Prediction insulation model (PIM)

The model could be modified to predict Hi-C data, which we called the “prediction insulation model” (PIM). For this purpose, we modeled the Hi-C count by a generalized linear model (Poisson regression):

6 $$\begin{aligned} \log \big ( \rm{E} \big [ \mathbf{y } | \mathbf{d },\mathbf{X } \big ] \big ) = \beta _0 + \beta _d\mathbf{d } + \mathbf{X } \varvec{\beta }_X \end{aligned}$$

Here, since we didn’t need to identify sharply the borders with L0 penalty, we could use directly the Poisson regression. PIM could be used to predict Hi-C data after chromosomal rearrangement. For this purpose, PIM was first trained using wild-type Hi-C data (no rearrangement). Then, the distance variable ($\mathbf{d }$) and the insulation variables ($\mathbf{X }$) were modified in a way to account for the chromosomal rearrangement. In the case of a deletion, the distance variable values were shrunk by the length of the deletion (producing a new distance variable noted $\mathbf{d }'$), and all insulation variables spanning the deletion were set to zero (producing new insulation variables $\mathbf{X }'$). In the case of an inversion, bins spanning the inversion were flipped and the distance variable and insulation variables were recomputed accordingly. The new variables ($\mathbf{d }'$ and $\mathbf{X }'$) together with the trained PIM model (with parameters ${\hat{\beta }}_0$, ${\hat{\beta }}_d$ and $\hat{\varvec{\beta }}_X$) were used to predict Hi-C data after rearrangement:

7 $$\log ({\text{E}}[{\mathbf{y}}{\text{|}}{\mathbf{d^{\prime}}},{\mathbf{X^{\prime}}}]) = \hat{\beta }_{{\text{0}}} {\text{ + }}\hat{\beta }_{{\text{d}}} {\mathbf{d^{\prime}}} + {\mathbf{X^{\prime}}} \hat{\varvec{\beta }}_X$$

## Results and discussion

### Identification of TAD borders and facilitators

**Fig. 1:**
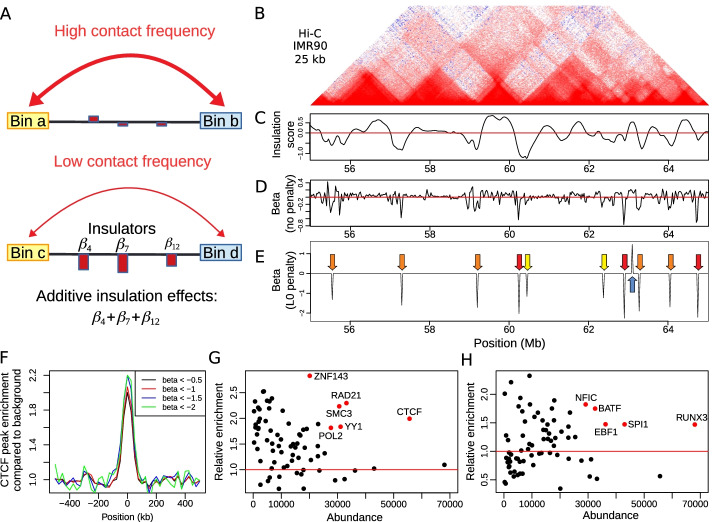
Illustration of the sparse insulation model (SIM) and identification of TAD borders and facilitators. **A** Schema representing the insulation effects modeled by SIM on long-range contacts between two bins (two loci), such as between an enhancer and a promoter. **B** Hi-C heatmap from IMR90 cells at 25 kb resolution. **C** Insulation score. **D** SIM beta (no penalty). **E** SIM beta (L0 penalty). **F** CTCF enrichment profile depending on border strength (beta). **G** DNA binding protein enrichment compared to background for several proteins at TAD borders identified by our model (negative betas) depending on protein abundance. **H** DNA binding protein enrichment compared to background for several proteins at TAD facilitators identified by our model (positive betas) depending on protein abundance

We proposed the sparse insulation model (SIM) to estimate the insulating/facilitating effects of genomic loci on long-range interactions (Fig. 1A). SIM required only one parameter, the maximal distance between two bins from the Hi-C matrix, which we set here to bin size $\times 10$ in order to reduce computational burden. We illustrated the model with high-depth Hi-C data at 25 kb resolution from human IMR90 lung cells, whose TADs could be easily visualized. We plotted the example of a 10-Mb-long genomic region of chromosome 1 (Fig. 1B). We first computed the insulation score (IS) to identify loci of high insulation. The insulation score is a standard measure reflecting the aggregate of interactions occurring across each interval. It is often used by experimentalists because of its simple and quantitative interpretation: the lower, the higher the insulation effect of the loci on overlapping contacts [8]. We observed peaks of negative IS, reflecting the presence of TAD borders with varying strengths (Fig. 1C). Alternatively, IS also revealed regions facilitating long-range contacts (score above zero).

Using SIM, we estimated instead sparse insulation scores (beta parameters). For a bin *i*, the $\beta _{x_i}$ parameter has a nice and intuitive interpretation: it is the insulation score, after accounting for the insulating/facilitating effects of the other bins. If no penalty is used to learn beta parameters, the betas correspond to a relative score (Fig. 1D). Using this relative score, we observed sharp peaks instead of wide valleys with the standard IS which prevented accurate location of TAD borders. Moreover, if an L0 penalty is used, then the regression leads to a sparse estimation of the insulation score. This helped to identify the exact location of bins with insulating/facilitating effects (Fig. 1E), in contrast to IS. In SIM, a negative beta value ($\beta _{x_i}<0$) reveals an insulation effect on long-range contacts (the bin is an insulator). Conversely, a positive beta value ($\beta _{x_i}>0$) shows a facilitating effect on contacts (the bin is a facilitator). A null beta value ($\beta _{x_i}=0$) means that the bin has no effect on contacts.

In the genomic region, SIM could detect ten TAD borders (${\hat{\beta }}<0$). Using SIM, TADs could be simply defined as regions in-between two consecutive TAD borders. Visual inspection of the Hi-C matrix clearly revealed that our TAD identification was relevant (Fig. 1B). Moreover, SIM could identify TAD borders with varying strengths. We found three strong TAD borders (${\hat{\beta }} < -2$; red arrows), five moderate TAD borders ($-1.2< {\hat{\beta }} < -2$; orange arrows) and two weak TAD borders (${\hat{\beta }} \approx -1.1$; yellow arrows). Moreover, the model uncovered one region with facilitating effects (${\hat{\beta }}>0$; blue arrow).

We then looked at the enrichment of the CTCF protein, a major 3D genome organizer, at TAD borders over the whole genome depending on the beta value. Here, we used GM12878 Hi-C data for which there are ChIP-seq data for a very large number of proteins, which helped us to comprehensively assess the role of DNA-binding proteins (see bellow). Overall, we found a strong two-fold enrichment of CTCF at TAD borders (Fig. 1F). Moreover, we observed that stronger TAD borders presented higher CTCF enrichment (2-fold for ${\hat{\beta }} < -0.5$; 2.2-fold for ${\hat{\beta }} < -1.5$), meaning that border strength estimated by SIM scaled accordingly with CTCF presence. Then, we evaluated enrichment for all available protein binding ChIP-seq data, and observed as previously shown the highest enrichments for CTCF, RAD21, SMC3, ZNF143, YY1 and POL2 (Fig. 1G) [2, 35, 36]. SIM could also identify regions facilitating contacts *e.g.* regions with ${\hat{\beta }}>0$ (we called “TAD facilitators”), unlike most TAD detection tools. IS could also detect facilitators, but without accurate location, thereby preventing enrichment analysis. Using SIM, we found that lymphocyte transcription factors (TFs) BATF, EBF1, NFIC, RUNX3 and SPI1 were enriched at such facilitator regions (Fig. 1H). Such high enrichment revealed that TAD facilitators were indeed biologically meaningful regions.

Thus, we could conclude that SIM had an intuitive interpretation in terms of insulating/facilitating quantitative effects, which could also sharply identify TAD borders unlike the insulation score. Moreover, our model could accurately identify a novel class of 3D elements that we called TAD facilitators, which were highly enriched in cell specific TFs.

### Performance and comparison with state-of-the-art tools

**Fig. 2:**
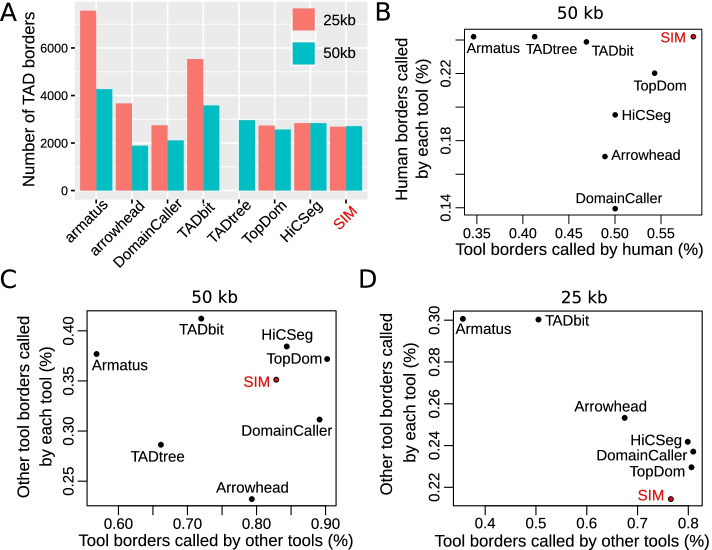
Comparison with existing TAD calling methods using GM12878 Hi-C dataset. **A** Number of TAD borders called by each method (50 kb resolution). **B** TAD border prediction concordance with manual annotation from [32] (50 kb). **C** TAD border prediction concordance between methods (50 kb). **D** TAD border prediction concordance between methods (25 kb)

SIM was very accurate to identify TAD borders. We compared it to 7 other algorithms including Armatus, Arrowhead, DomainCaller, TADbit, TADtree, TopDom, HiCseg using human GM12878 Hi-C data as from [32] (Fig. 2). At both 25 kb and 50 kb, SIM identified a small number of TAD borders (2691 and 2711, respectively), such as HiCSeg (2835 and 2835, respectively) and TopDom (2738 and 2568, respectively) (Fig. 2A). Conversely, Armatus identified much more TAD borders (7567 and 4265, respectively) (Fig. 2A). Overall, we found that the number of borders identified by SIM (as well as HiCseg and TopDom) was only slightly impacted by Hi-C data resolution, unlike for the other algorithms. We also compared the TAD borders identified by SIM for different normalizations of the Hi-C data (Knight-Ruiz (KR) [30], iterative correction and eigenvector decomposition (ICE) [37] and square root vanilla coverage (VC SQRT) [38]), and globally found similar results at 50 kb resolution (Additional file 1: Figure S2). We then compared TAD border prediction concordance with manual annotation of TADs at 50 kb from [32] (Fig. 2B). These manually annotated TADs represented an external assessment which was designed not to favor any tool. We found that $58.5\%$ of borders predicted by SIM were also found by manual annotation, which ranked first SIM. Moreover, SIM was able to detect $24.2\%$ of manually annotated borders. In comparison, the large numbers of TAD borders detected by Armatus (>4000 at 50 kb) or TADbit (>3500 at 50 kb) were proportionally less confirmed by manual annotation ($34.6\%$ and $46.9\%$, respectively).

We then assessed TAD border prediction concordance between the different tools. At 50 kb, $82.8\%$ of borders detected by SIM were also identified by the other tools, and $35.2\%$ of other tools’ borders were called by SIM, which was similar to the top tools, HiCSeg and TopDom (Fig. 2C). At 25 kb, $76.5\%$ of borders detected by SIM were also identified by the other tools, and $21.4\%$ of other tools’ borders were called by SIM, which was similar to HiCSeg, TopDom and DomainCaller (Fig. 2D). Thus, SIM ranked among the best tools to predict TAD border. Meanwhile, SIM was relatively fast and memory efficient. For chromosome 1 with 25 kb resolution and considering a maximal distance of 250 kb, SIM ran in only 151 seconds for one core and around 6.9 Gb.

### Identification of novel borders during cell differentiation

The 3D genome is dynamic, especially during the developmental process, and global reorganization was previously reported during differentiation [29]. However, very few methods were developed for differential analysis of TADs [20, 22]. Using our versatile regression framework, we could easily implement differential TAD analysis in order to identify novel TAD borders, or alternatively depleted TAD borders, during cell differentiation. For this purpose, interaction terms were added in the model to account for differential insulation effects depending on the cell type. We called this model the differential insulation model (DIM). The corresponding interaction betas were then used to assess differential TAD border strength.

**Fig. 3:**
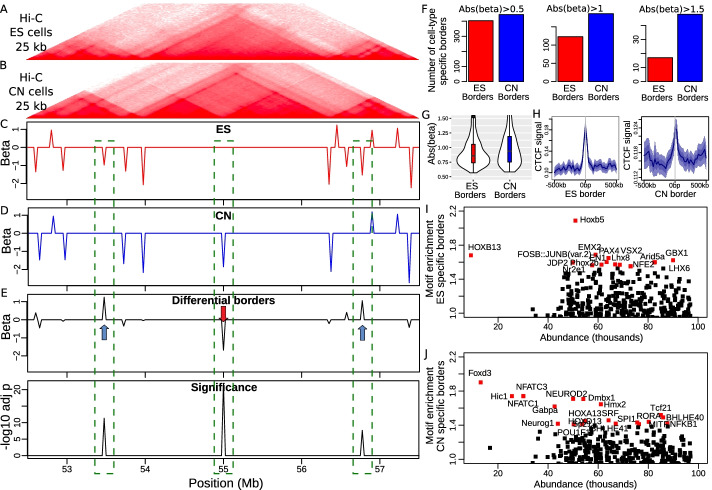
Differential analysis of TAD borders with the Differential Insulation Model (DIM). **A** Hi-C heatmap in mouse embryonic stem (ES) cells. **B** Corresponding Hi-C heatmap in cortical neuron (CN) cells. **C** Identification of TAD borders in ES cells (spase insulation model beta is plotted). **D** Identification of TAD borders in CN cells (spase insulation model beta is plotted). **E** Identification of differential TAD borders (DIM beta is plotted). For each beta, an adjusted *p*-value is plotted to show significance. **F** Number of cell-type specific borders, for varying differential border strengths. **G** Absolute value of beta between CN specific borders and ES specific borders. **H** CTCF enrichment at CN specific borders compared to ES specific borders. **I** DNA-binding protein motif enrichment (fold-change) at CN specific borders. **J** DNA-binding protein motif enrichment (fold-change) at ES specific borders

To illustrate differential analysis, we studied mouse embryonic stem cells (ESs) differentiation into cortical neurons (CNs) using ultra-deep coverage Hi-C, where novel TAD borders were shown to colocalize with developmental genes that were activated [29]. We first focused on a 5-Mb-long genomic region of chromosome 18 around the developmental gene *Zfp608*. In ES cells, we observed a big TAD in the middle of the Hi-C map (Fig. 3A, C). In CN cells, this big TAD was split into two new TADs separated by a novel border located at 55 Mb overlapping the gene *Zfp608* (Fig. 3B, D). Using the two Hi-C maps, DIM accordingly identified a strong and significant differential TAD border at 55 Mb (${\hat{\beta }} \approx -1.8$, $p<10^{-70}$; blue arrow; Fig. 3E), reflecting TAD split during differentiation. Moreover, DIM could also reveal less obvious differences in border strength. In particular, DIM detected two smaller differential TAD borders (${\hat{\beta }}<1.2$, $p<10^{-8}$; red arrows), which corresponded to borders present in ES cells and lost in CN cells.

We then ran differential analysis by DIM genome-wide. We observed a higher number of TAD borders after differentiation (fold-change = 1.1; Fig. 3F, left), meaning that new TADs were created after differentiation. If we only considered strong TAD borders, we observed an even larger number of TAD borders after differentiation (fold-change = 1.51 for abs(beta)$>1$; fold-change = 2.82 for abs(beta) $>1.5$). Moreover, the absolute values of DIM betas in CN were significantly higher than in ES (fold-change = 1.11, *p*-value = 0.01; Fig. 3G), suggesting that those new TADs were particularly strong and insulated. We then compared CTCF enrichment at CN-specific borders and ES-specific borders (Fig. 3H). We found that although CTCF was very enriched at ES borders (fold-change = 1.64), it was far less enriched at CN borders (fold-change = 1.07), suggesting that the novel TAD borders were maintained by other factors than CTCF. It was previously showed that novel TAD borders located to neural transcription factors Pax6, NeuroD2, and Tbr1 [29]. However, their analysis was limited by available ChIP-seq data. Here, instead, we systematically assessed the enrichment of 579 protein binding DNA motifs at novel CN borders (Fig. 3I). We found a tremendous amount of motifs enriched at novel borders. All enriched motifs were known neural TFs, including Foxd3, NFATC3, NEUROD2, HiC1, Dmbx1, Hmx2 and NFATC1. This result suggested that chromatin was reorganized due to not only Pax6, NeuroD2, and Tbr1, but also to numerous other TFs involved in neural differentiation. In comparison, ES borders were strongly enriched in known stem cell TFs, such as Hoxb5, EMX2, PAX4. Thus, we could conclude that cell type specific TFs played a major role in reshaping the genome in 3D during differentiation.

### Predictions of Hi-C data after chromosomal rearrangements

Our versatile regression framework could also be used to faithfully model the 3D genome and predict Hi-C data. In particular, predicting the effects of chromosomal rearrangement on 3D genome is an important challenge, since 3D genome alteration can impact essential cellular processes such as enhancer-promoter transcriptional regulation. However, until now, only few methods were developed for this task. Hence, we assessed the ability of the model to predict Hi-C data after chromosomal rearrangement. In this case, we called this model the prediction insulation model (PIM). For this purpose, PIM was trained on wild-type (WT) Hi-C data, producing a model with parameters ${\hat{\beta }}_0$, ${\hat{\beta }}_d$ and $\hat{\varvec{\beta }}_X$. Then, in the PIM model, the distance variable ($\mathbf{d }$) and the insulation variables ($\mathbf{X }$) were modified in a way to account for the chromosomal rearrangement. For instance, in the case of a deletion, the distance variable values were shrunk by the length of the deletion (producing a new distance variable noted $\mathbf{d }'$), and all insulation variables spanning the deletion were set to zero (producing new insulation variables $\mathbf{X }'$). The new variables together with the trained PIM model were used to predict Hi-C after deletion.

**Fig. 4:**
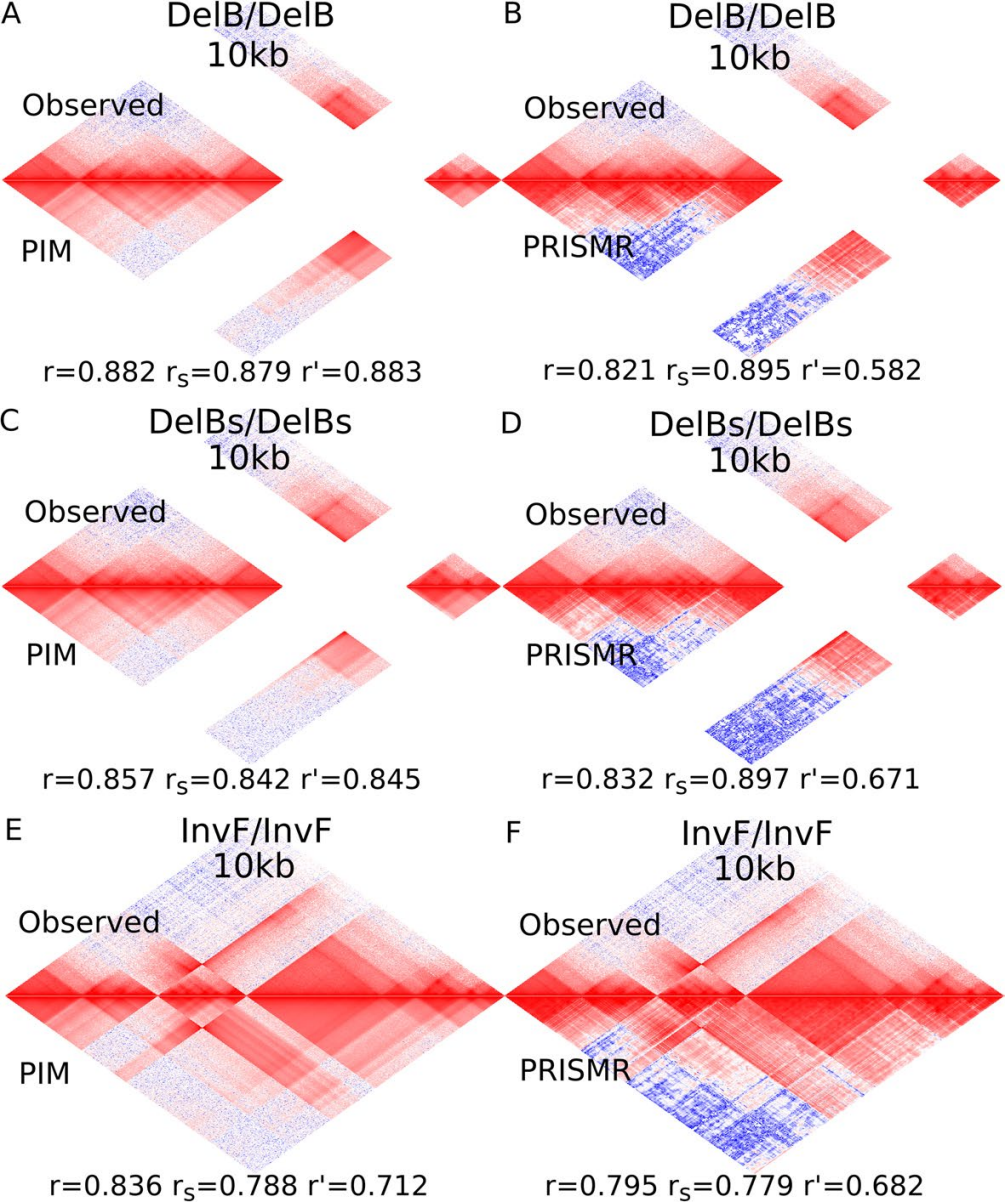
Prediction insulation model (PIM) predicts rearranged 3D genome with high accurary and comparison with PRISMR using mouse data from [23]. Models were trained using wild-type Hi-C data to predict rearranged Hi-C data (for PRISMR, we used predictions provided by the authors). **A** PIM prediction for DelB/DelB genotype and comparison with observed data. **B** PRISMR prediction for DelB/DelB genotype. **C** PIM prediction for DelBs/DelBs genotype and comparison with observed data. **D** PRISMR prediction for DelBs/DelBs genotype. **E** PIM prediction for InvF/InvF genotype and comparison with observed data. **F** PRISMR prediction for InvF/InvF genotype

PIM prediction accuracy was assessed using 10 kb resolution capture Hi-C experiments performed in E11.5 limb buds from WT and mutant mouses with a deletion or an inversion [23]. For the DelB/DelB mutant (homozygous deletion), we found very accurate Hi-C data predictions as compared to observed data in the mutation mouse (Fig. 4A). Most notably, PIM was able to finely model the distance effect, the numerous TADs, but also the complex hierarchies of TADs. Prediction accuracy was very high as measured by Pearson correlation between log-counts $r=0.882$ and Spearman correlation between counts $r_s=0.879$ (Fig. 4A). In comparison, the state-of-the-art model PRIMSR achieved comparable performance in terms of Pearson and Spearson correlations ($r=0.821$, $r_s=0.895$; Fig. 4B). But, when distance effect was removed using stratum adjusted correlation in order to only capture the biological variability, PIM performed better than PRISMR (PIM: $r'=0.883$ and PRISMR: $r' = 0.582$; Fig. 4A, B), reflecting its better ability to model biological variability underlying TADs and sub-TADs. We next compared PIM and PRIMSR using other mouse mutants. For the DelBs/DelBs mutant, we also found that PIM and PRISMR achieved similar performance in term of *r* and $r_s$ (PIM: $r=0.857$, $r_s=0.842$; PRISMR: $r=0.832$, $r_s=0.897$; Fig. 4C, D), but PIM predictions compared favorably in term of biological variability with $r'$ (PIM: $r=0.845$; PRISMR: $r=0.671$; Fig. 4C, D). Lastly, we predicted data for an inversion (InvF/InvF). As for deletions, we found that PIM yielded better predictions than PRISMR in term of biological variability with $r'$.

## Conclusion

In this article, we propose a versatile regression framework for Hi-C data analyses. Our framework was designed for TAD identification (SIM model), but also differential analysis (DIM model) and Hi-C data predictions after chromosomal rearrangement (PIM model). First, SIM accurately detected TAD borders in a quantitative manner, and was ranked among the top TAD callers when comparing with state-of-the-art methods on an unbiased dataset. Moreover, SIM also identified a novel class of elements we called facilitators which facilitated long-range contacts as opposed to borders, and were shown to be associated with specific transcription factors. Second, DIM identified novel borders during neuronal differentiation. Such novel borders were particularly enriched for other factors than CTCF, in particular, numerous transcriptional factors specific to neurons including Foxd3, NFATC3, NEUROD2, HiC1, Dmbx1, Hmx2 and NFATC1. In comparison, ES specific borders were enriched in stem cell TFs. Third, PIM accurately predicted rearranged 3D genome in mouse mutants, when trained with wild-type Hi-C data. Such approach is very promising to assess the impact of chromosomal rearrangements on the 3D genome. Moreover, PIM compared favorably with state-of-the-art PRISMR in terms of biological variability captured by Hi-C data.

There are several limitations of the proposed framework. First, the proposed framework is designed for the analysis of bulk Hi-C data, *i.e.* data from a population of cells. However, single-cell experiments are getting widely used in 3D genome studies, and necessitate the development of new tools. The proposed framework must be further extended for data that are too sparse, which is the case for single cell data. The use of an empirical Bayes approach to estimate regression betas across cells might be a elegant solution for this purpose. Second, the same framework can be further extended for other Hi-C data analysis tasks. For instance, the regression can be used to infer frequently interacting regions (FIREs) and differential FIREs from Hi-C data [39]. Third, variable selection for the SIM model is based on best subset selection using L0Learn R package. However, one problem is that L0Learn cannot work with more than 5000 variables on a standard computer, and for the largest chromosomes, prefiltering is done using lasso regression and a threshold of $|{\hat{\beta }}_{x_i}|>0.2$ to sufficiently reduce the number of variables for processing. However, this prefiltering might affect best subset selection. Other prefiltering approaches not relying on an arbitrary thresholding can be used instead. For instance, knockoff can be used for removing unnecessary variables while controlling the false discovery rate (FDR) [40]. Alternatively, bootstrap stability investigation can be used [41]. Fourth, SIM is methodologically similar to other TAD callers based on the computation of a linear score such as TopDom [10] or those based on statistical models of the interaction distributions such as HiCseg [11]. We thus expect SIM to call similar TAD borders (performances between SIM, TopDom and HiCseg were similar, Fig. 2). But SIM is very different from other TAD callers based on clustering [14–16] or graphs [17–19], and thus SIM is more likely to miss those TADs. Fifth, compared to other TAD callers, SIM is conservative for the detection of TAD borders, meaning that fewer but correct TADs were called rather than many TADs including a few false positives. This stringency is related to the use of best subset selection. The use of other variable selection procedures could be investigated to assess if more TAD borders could be identified.

## Supplementary Information

**Additional file 1**.** Figure S1**. Comparison of betas between SIM with prefiltering by lasso regression and SIM without prefiltering.** Figure S2**. Comparison of TAD borders identified by SIM for different normalizations of the Hi-C data (Knight-Ruiz (KR)), iterative correction and eigenvector decomposition (ICE) and square root vanilla coverage (VC SQRT) at 50 kb resolution.

## Data Availability

An R package called “TADreg” was developed and is available at: https://github.com/raphaelmourad/TADreg.
