# Supplementary material for: TADreg: a versatile regression framework for TAD identification, differential analysis and rearranged 3D genome prediction
Source: BMC Bioinformatics. 2022 Mar 2;23:82. doi: 10.1186/s12859-022-04614-0 (PMC8892791; doi:10.1186/s12859-022-04614-0)
Supplement: Supplementary file 1 — Additional file 1. Figure S1. Comparison of betas between SIM with prefiltering by lasso regression and SIM without prefiltering. Figure S2. Comparison of TAD borders identified by SIM for different normalizations of the Hi-C data (Knight-Ruiz (KR)), iterative correction and eigenvector decomposition (ICE) and square root vanilla coverage (VC SQRT) at 50 kb resolution. [file 12859_2022_4614_MOESM1_ESM.pdf]

Supplementary Figures

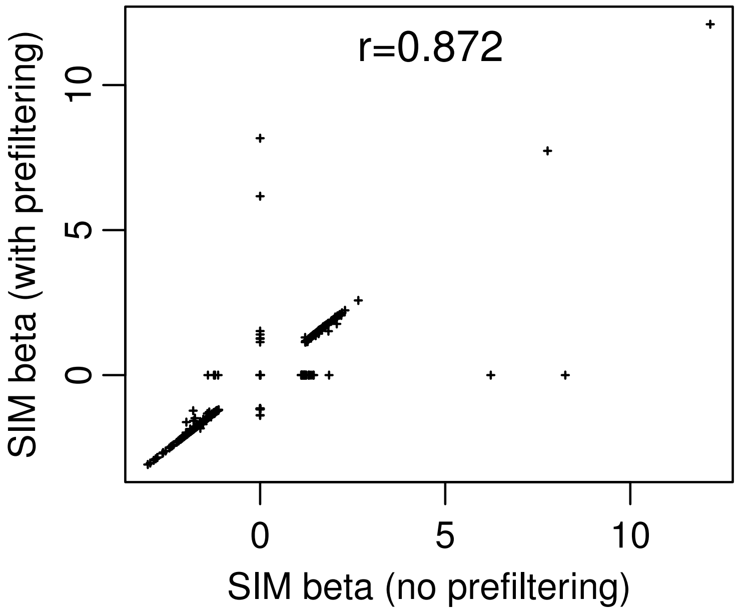

Supp Fig S1: Comparison of sparse insulation model (SIM) beta estimation with and without prefiltering of variables by lasso regression.

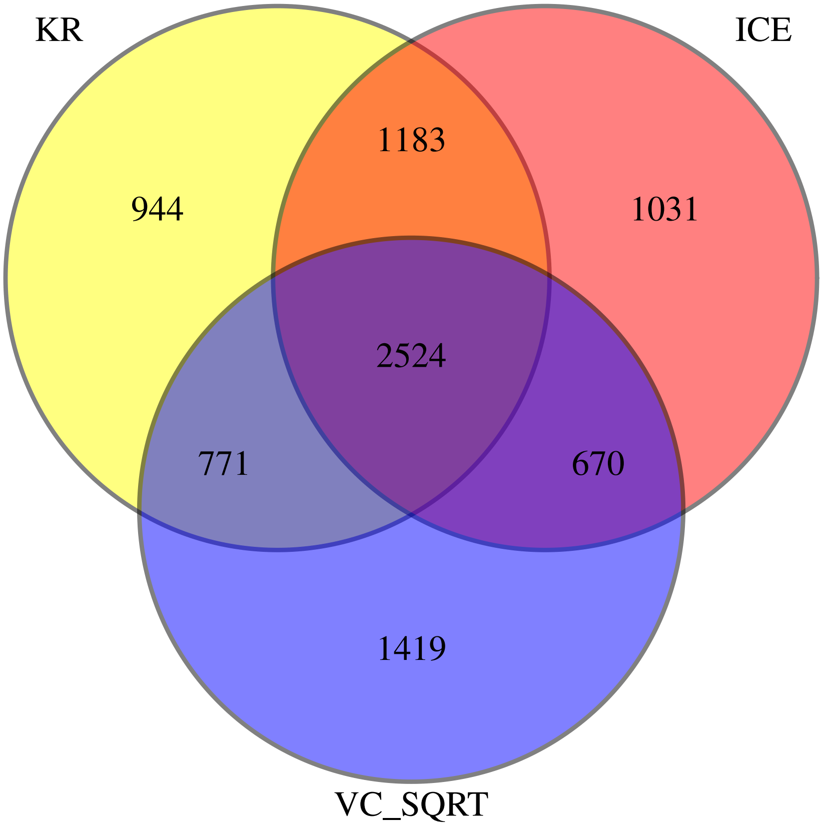

Supp Fig S2: Comparison of TAD borders identified by sparse insulation model (SIM) for different Hi-C data normalizations: Knight-Ruiz (KR), iterative correction and eigenvector decomposition (ICE) and square root vanilla coverage (VC SQRT).
